# Curve-fitting alone cannot validate neutral theory

**DOI:** 10.1073/pnas.2412160122

**Published:** 2025-03-03

**Authors:** Evan C. Johnson

**Affiliations:** ^a^Department of Mathematical and Statistical Sciences, University of Alberta, Edmonton, AB T6G 2R3, Canada

## 1. Comment

In a recent paper, Saulsbury et al. (1) show that neutral theory (NT) accurately predicts the extinction rates of graptoloids, an ancient clade of planktonic animals. NT is controversial because it assumes that all species share the same ecological niche, attributing differences in abundance to random variation in births and deaths. Saulsbury et al. argue for NT by showing that it predicts an elevated extinction rate for young species, whereas Van Valen’s Red Queen (RQ) hypothesis does not. There are two problems with this comparison. First, it is unclear whether higher extinction rates for young species are a general pattern (references in ref. 2). Second, while Van Valen argued forcefully for age-independent extinction rates, the RQ hypothesis itself is compatible with an elevated extinction rate for young species; mechanisms include pseudoextinction (young species transform via anagenesis), selection bias [species with high extinction rates are short-lived (3)], and the vulnerability of young populations due to their limited geographic extent.

More importantly, Saulsbury et al. suggest that NT can be validated by curve-fitting, despite NT’s legacy showing the opposite. The original evidence for NT, left-skewed distributions of species abundance, turned out to be consistent with numerous nonneutral processes, including dispersal dynamics and niche-allocation models (e.g., ref. 4). This historical episode demonstrates that curve-fitting provides extremely weak evidence: Most theories can predict simple patterns, so simple patterns do not imply a single theory (5).

To illustrate the principle, I propose a model with nonoverlapping niches. Species grow exponentially with the per-capita growth rate *r*, are bounded below carrying capacity *K*, and are subject to environmental noise scaled by *σ*. The dynamics can be written as a stochastic differential equation:

[1] dX=rXdt+σXdW,

where *X* is population abundance and *dW* is the Brownian motion increment. Intraspecific density dependence is enforced by a reflecting boundary at *X* = *K*, making this equation qualitatively similar to, but simpler than, the more conventional logistic model.

**Fig. 1.**
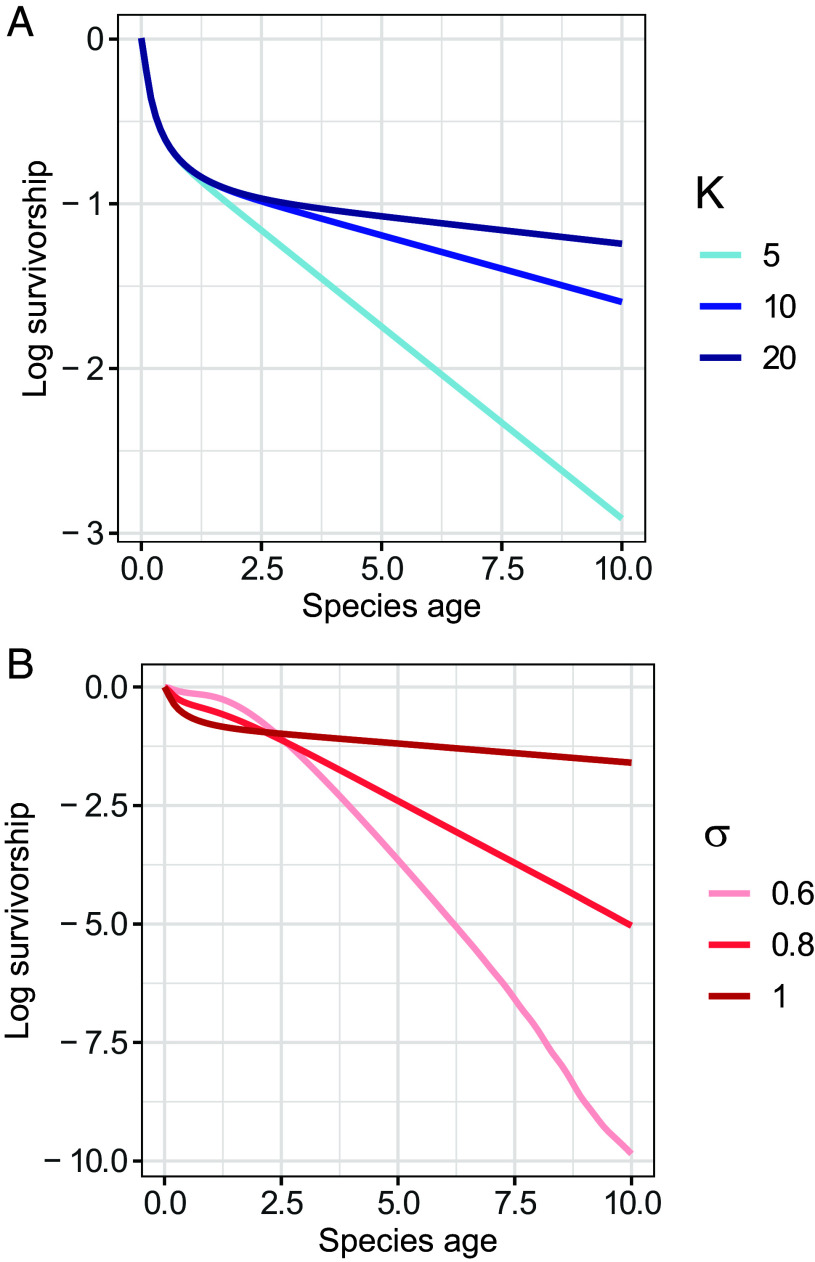
A simple model without interspecific competition can generate a variety of relationships between species age and extinction rates. The units of time/age are arbitrary. (*A*) *K* varies, with *r* = 1 and *σ* = 1. (*B*) *σ* varies, with *r* = 1 and *K* = 10.

My alternative model produces flexible survivorship curves, including elevated extinction risk for young species (Fig. 1). Further, extinction in this model involves sustained environmental change and rapid population collapse (6), both features of real-world population extinction (7).

A holistic evaluation of NT should consider other predictions, the validity of assumptions, and how the theory withstands deviations from these assumptions. NT predicts unrealistically high speciation rates and overly stable population abundances (8). The core assumption of neutrality—ecological equivalence—is not true in any known case (9). NT collapses under small deviations from strict neutrality, as the dominant species rapidly excludes others.

Despite the shortcomings of NT, the importance of ecological drift should not be dismissed. A semirecent study (10) demonstrated that NT, when augmented with environmental stochasticity, could explain community patterns in tropical trees. The problematic nature of curve-fitting was circumvented by directly deriving parameters from data and validating the model against multiple patterns. Strict neutrality was replaced with a fluctuation-based coexistence mechanism, the storage effect, thus marking an advance toward the synthesis of neutral and niche theories. By not considering other critical evidence and relying on curve-fitting, Saulsbury et al. jeopardize the integration of neutral and niche theories, a project which they aim to advance.
